# Ultrasonic-assisted supercritical-CO_2_ electrodeposition of Zn-Co film for high-performance corrosion inhibition: A greener approach

**DOI:** 10.1016/j.ultsonch.2021.105463

**Published:** 2021-01-14

**Authors:** Sabarison Pandiyarajan, Muthusankar Ganesan, Ai-Ho Liao, Shobana Sebastin Mary Manickaraj, Sheng-Tung Huang, Ho-Chiao Chuang

**Affiliations:** a Department of Chemical Engineering and Biotechnology, National Taipei University of Technology, Taipei 10608, Taiwan; b Department of Mechanical Engineering, National Taipei University of Technology, Taipei 10608, Taiwan; c Graduate Institute of Biomedical Engineering, National Taiwan University of Science and Technology, Taipei, Taiwan; d Department of Biomedical Engineering, National Defense Medical Center, Taipei, Taiwan; e Department of Industrial Chemistry, Alagappa University, Karaikudi 630001, Tamil Nadu, India

**Keywords:** Electrodeposition, Ultrasonication, Supercritical-CO_2_, Microhardness, Corrosion

## Abstract

The ultrasonic-assisted electrodeposition process significantly improves the mechanical and electrochemical properties. Meanwhile, supercritical fluid technology also enhances the electrodeposition process with increased benefits, such as low surface tension, permeability, high diffusivity, and density, which improves the surface quality through grain refinement. In this study, Zn-Co films were prepared using the ultrasonic**-**assisted supercritical-CO_2_ (US-SC-CO_2_) electrodeposition approach, and its pressure effect on the film was evaluated. The films were also prepared by the conventional and typical supercritical-CO_2_ (SC-CO_2_) methods for a comparison study. All the prepared films were characterized by morphological studies, elemental composition, crystal structure orientation, and microhardness tests. Later, the fabricated films were examined by potentiodynamic polarization technique and electrochemical impedance technique (EIS) with 3.5 wt.% NaCl solution for corrosion evaluation. Based on results, Zn-Co film prepared through the US-SC-CO_2_ process shows a spherical nodule like structure with reduced grain size and significantly enhanced hardness property. In XRD studies, the shift in diffracted peak’s position reveals the increased proportion of Co ions. Further, EDX results also confirm the same with the characteristic peaks. Finally, compared to the other methods, the corrosion resistance was more efficient in the US-SC-CO_2_ process by 73.75%.

## Graphical abstract

**Figure:**
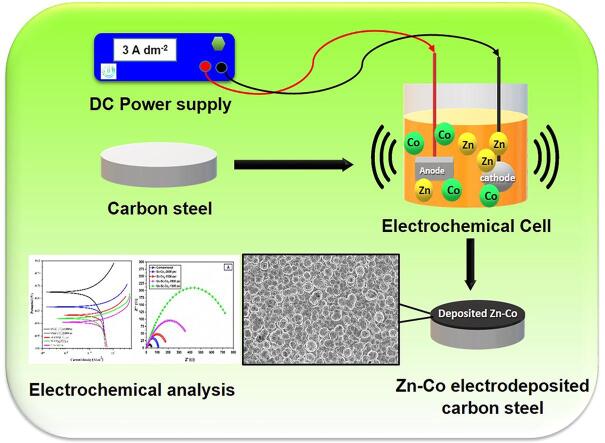

## 1 Introduction

Owing to the outstanding physical and mechanical qualities, carbon steel has been heavily cast-off in building, petroleum oil, and computer manufacturing industries of all sorts. However, its active chemical natures were easily affected by conditions that included oxygen and chlorine. Thus, the significant drawback severely affected the lifetime of the commodity in the product and caused a substantial economic loss [1]. Thanks to thin films and their exclusive properties with extensive uses in science and technology, which drives the manufacturing of nano-crystalline coatings that had a superior value over the past [2]. Various techniques such as sol-gel, a self-assembled monolayer (SAM), vapor deposition, sputtering, and electrodeposition have been developed to produce a thin layer of metal coating. Among them, electrodeposition is one of the most prominent techniques for creating the thin layer of nano-crystalline precious metal deposition on the cheapest metal surface at an affordable cost [3], [4], [5], [6], [7].

Because of the excellent physical properties and economic performance, zinc (Zn) has been most commonly used as a sacrificial and electrical barrier material to defend ferrous material with crucial advantages in various applications [8], [9], [10]. Moreover, Zn-composite with transition metals, especially Ni, Co, Fe, has been of great interest due to their strong ability to improve corrosion resistance relative to solitary zinc coatings [11]. Zinc and cobalt are precious metals with superior properties to create alloy deposition, and it also appears to be a suitable replacement for harmful chromium and cadmium coatings. Many published innovations indicate that the alloys dependent on cobalt exist in superior nature to others. Cobalt ions incorporation into the Zn matrix through the electrodeposition composite coating significantly improves corrosion resistance with superior impact on tensile strength and rigidity [12]. In 2008, Mouanga et al. reported coumarin’s influence on the zinc–cobalt alloy derived from an acid chloride electrolyte [13]. In 2017, Azizi et al. reported that the higher concentrations of cobalt ions in Zn-Co electrodeposition acquired improved corrosion resistance [14].

Indeed, to maintain the purity of electrolytes and to reduce environmental pollution, supercritical carbon-di-oxide (SC-CO_2_) is used as a stable emulsion surfactant. The advanced green electroplating process with SC-CO_2_ has numerous advantages such as non-toxic, improved adhesion strength, smoother coating surface, comparatively lower critical point than others, and readily available from many industrial by-products [15], [16], [17]. Sone et al. discovered the pressure effects on grain size in SC-CO_2_ electroplating and originated the least grain size was detected at high pressure (15 MPa). Besides, Cu pillars without cavities were accomplished efficaciously via the supercritical electroplating process [17], [18], [19]. Our research group also performed studies on heat treatment's effect before and after on supercritical electroplating to acquire nickel-filled TSVs and inspected its vacuum sealing and electrical resistance [20]. During the electrodeposition reaction, emulsified dense carbon-di-oxide is mixed to the electrolyte, rapidly eliminate the hydrogen evolution by creating more microbubbles and accelerate the speed of metal ions through increasing the chemical kinetic reaction [21], [22], [23]. Moreover, the utilization of SC-CO_2_ is an excellent alternate to toxic organic solvent, also functional recovery from the industrial release of CO_2_ to the atmosphere

Numerous application techniques were commonly strived to improve the alloy’s mechanical and electrochemical behavior. Ultrasonication is a superior application technique that creates heavy electrolyte irradiation resulting in substantial electronic, physical, and chemical disruptions by acoustic cavitation [24], [25]. Ultrasonic stimulation enables the mass transfer of metal ions to the electrolyte through the action of cavitation implosion, micro-jetting, radiation pressure impact in the electrolyte bath to intensify the electrochemical (micro) reactions [26]. According to the Pollet et al. report, if the ultrasonic supported electrodeposition process’ conditions were appropriately adopted, it could expressively improve the quality of deposited metals [27]. The ultrasound-assisted with conventional electrodeposition method has several beneficial effects, such as increases the active surface area, adhesion strength, and crystallite size, and diminishes the compressive pressure, which tends to increase microhardness and electrochemical behavior with decreased grain size [28], [29], [30], [31]. In 2020, Ridosic et al. report the benefit of Zn-Co coating with ultrasound and corrosion stability [32]. Kobayasi et al. report that the frequency in the lower range could play a significant role in improving charge transfer reaction and altering the nucleation growth. The effect was present in the following order: no effect = silent conditions < 100 kHz < 28 kHz < 45 kHz = highest effect [33]

Nonetheless, SC-CO_2_ aided in the Zn-Co electrodeposition is no longer released with the integrated ultrasonic method. With ultrasonic agitation in the SC-CO_2_ process, a more effective emulsion effect can be achieved, significantly eliminating the hydrogen adsorption on the cathode, which was typically found in conventional methods. In some situations, surfactants may be unacceptable because they could impact the film’s quality, and the electrolyte may be difficult to process or reclaim afterward. Also, ultrasound irradiation to the supercritical condition produces high-energy bubble bursting, leads to the periodic plastic effect that improves the ion deposition compatibility through the leveling effect [34], [35], [36].

From the light of the above-mentioned facts, the present study focuses on the fabrication of Zn-Co composite films by without additives or any surfactants. The SC-CO_2_ replaces the role of surfactant. Therefore, the investigation is based on the competition control over the manufacturing of Zn-Co films. The mechanical and electrochemical properties of the electrodeposited Zn-Co alloy films were carefully researched. The results revealed that the film prepared using the US-SC-CO_2_ method of electrodeposition showed the best performance than other methods.

## 2 Experimental

### 2.1 Materials

Zn-Co electrolyte was prepared with 0.1 M solution of zinc sulfate heptahydrate (ZnSO_4_·7H_2_O), 0.1 M solution of cobalt sulfate heptahydrate (CoSO_4_·7H_2_O), and 0.4 M solution of boric acid (H_2_BO_3_). Electrochemical studies were conducted with 3.5 wt.% sodium chloride (NaCl) solution for corrosion studies. These chemicals were purchased from M/s Sigma Aldrich and used as received. Zn-Co metals precursors were provided by Zn-Co sulfate with boric acid supported electrolyte. Additives and surfactants were not added to the electrolyte to maintain the electrolyte purity.

### 2.2 Electrodeposition methods

The electrodeposition reactions were carried out in three different methods: conventional, SC-CO_2_, and US-SC-CO_2_ approaches. All the reactions were conducted in a 100 ml reaction cell by the galvanostatic way with a circular carbon steel sheet (2.27 mm^2^) acting as a cathode and rectangular cobalt bar (25 × 20 × 5 mm) acting as an anode. Before, the reaction substrate was exposed to the pretreatment process, such as grinding with 1500 grit emery sheet and polished with 1 µm of alumina. After that, the substrate was sonicated with ethanol and water for 15 mins to remove the impurities. Finally, the substrate was prepared to use in the reaction. The applied current density was 3 A/dm^3^, and the ambient working temperature is 50 °C for all the methods as mentioned earlier. In the SC-CO_2_ method, 1500 and 2000 psi were used as a supercritical pressure. The reactions were carried out in a stainless steel cell with a high pressured stainless steel chamber used as a supercritical working environment.

**Scheme 1:**
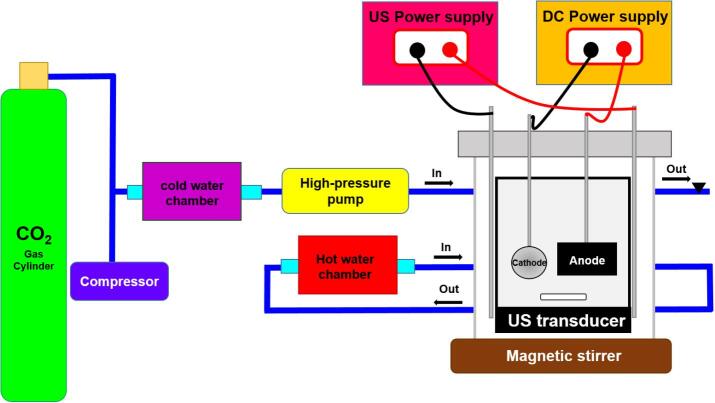
Schematic representation of high-pressure experimental arrangement.

Additionally, the stainless-steel reaction cell was modified with a circular piezoelectric transducer for the US-SC-CO_2_ electrodeposition method to produce ultrasonic irradiation to the reaction system. The modified circular piezoelectric transducer was generated at a constant 42 kHz frequency with an adjustable power supply. In this work, the ultrasonic irradiation was generated with 42 kHz/20 W cm^−2^. The schematic representation of the high-pressure experimental arrangement is illustrated in Scheme 1.

### 2.3 Characterization of Zn-Co film

The prepared Zn-Co composite film is probed by a thin-film X-ray diffraction study (Model- X’Pert Pro powered by pANalytical). From the main diffracted peak, the average grain size can be estimated using the Scherer equation (1),

(1) $$D_{\left(hkl\right)}=\frac{k\lambda }{\beta cos\theta }$$

Here, $D_{\left(hkl\right)}$ is the average grain size, k is shape constant (0.9), λ is the incident wavelength of x-ray (λ = 0.15405 nm), β is the full width half maximum (FWHM), θ is the incident angle of x-ray. The prepared films’ morphology is examined by a field emission scanning electron microscope (FESEM) (Model- sigma essential powered by Zeiss Microscopy). The hardness inspection of the prepared films is measured with the HM-113 by Mitutoyo Corp Vickers hardness machine by applying 100 g load with a dwell time of 10 s. The electrochemical studies are probed through Autolab PGSTAT302N electrochemical workstation by Metrohm. The corrosion analysis evaluation was conducted with potentiodynamic polarization scanning (PPS) and electrochemical impedance spectroscopy (EIS) with the immersion of 3.5 wt.% NaCl solution. The conventional three-electrode system was used for all the electrochemical studies. All the prepared films were served as a working electrode, Ag/AgCl as a reference electrode, platinum wire as a counter electrode.

## 3 Results and discussion

### 3.1 Film growth mechanism

According to the electrochemical reaction, when we apply the current to the electrochemical cell, the possible electrode reaction [4], [5], [12], [37] could be expressed as follow:

Cathode

(2) (i) Primary reaction: Zn(OH)_2_ + e^−^ → ZnOH + OH^−^

(3) ZnOH + e^−^ → Zn + OH^−^

(4) Co(OH)_2_ + e^−^ → Co(OH)^+^ + OH^−^

(5) Co(OH)^+^ + e^−^ → Co + OH^−^

(6) (ii) Secondary reaction: 4H^+^ + 2e^−^ → H_2_ ↑

(7) Anode: 8 OH^−^ + 8e^−^ → O_2_ ↑ + H_2_O

**Fig. 1:**
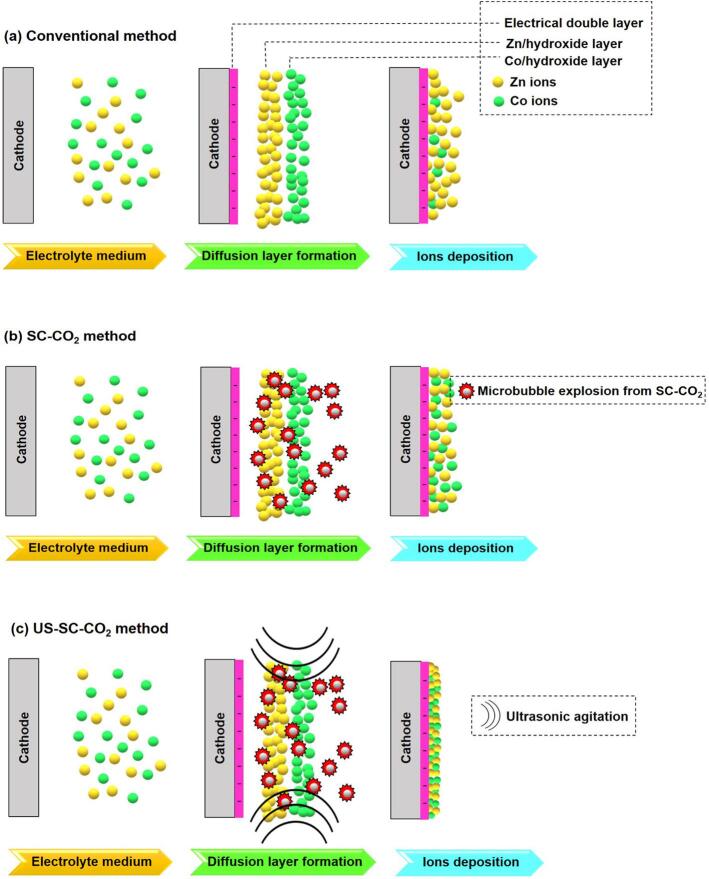
Schematic representation of possible film growth mechanism.

The possible film growth mechanism of Zn-Co composite film is schematically represented in Fig. 1. Initially, the Zn and Co ions were positively charged and randomly dispersed in the electrolyte bath. When the current is applied to the cell, the electric field is instantly formed between the anode and cathode. Then the applied current influences the metal ions to migrate towards the cathode. Where they get reduced to zero-valent atoms that yield strong adsorption on the surface of the cathode. Notably, the Zn ions are adsorbed more readily than Co ions due to having a lower standard reduction potential.

The co-deposition of Co metal ions is deemed an anomalous deposition, whereas the concentration of Zn ions exceeds, and the proportion of Co ions deposition surpassed its quantity in the electrolyte bath. This fascinating performance can be attributed to hydrogen evolution, which leads to an increase in the concentration of local hydroxyl ion (OH^−^). Thus, the OH^−^ ions provide a strong adsorption effect on existing metal ions to deposit on the substrate. With the help of these hydroxyl ions, Co gets incorporated into the Zn matrix [37]. In the SC-CO_2_ method, microbubble explosion significantly decreases the Zn(OH)_2_ layer’s density and allows more Co ions to reach the cathode. On the other hand, with the introduction of ultrasonic irradiation to the pressurized CO_2_ environment, the more intense cavitation effect enhances the emulsification process and resulting in “soft cavitation behavior,” which significantly reduces the hydrogen evolution and improve the leveling effect on the surface of the film.

### 3.2 Morphology of Zn-Co films

**Fig. 2:**
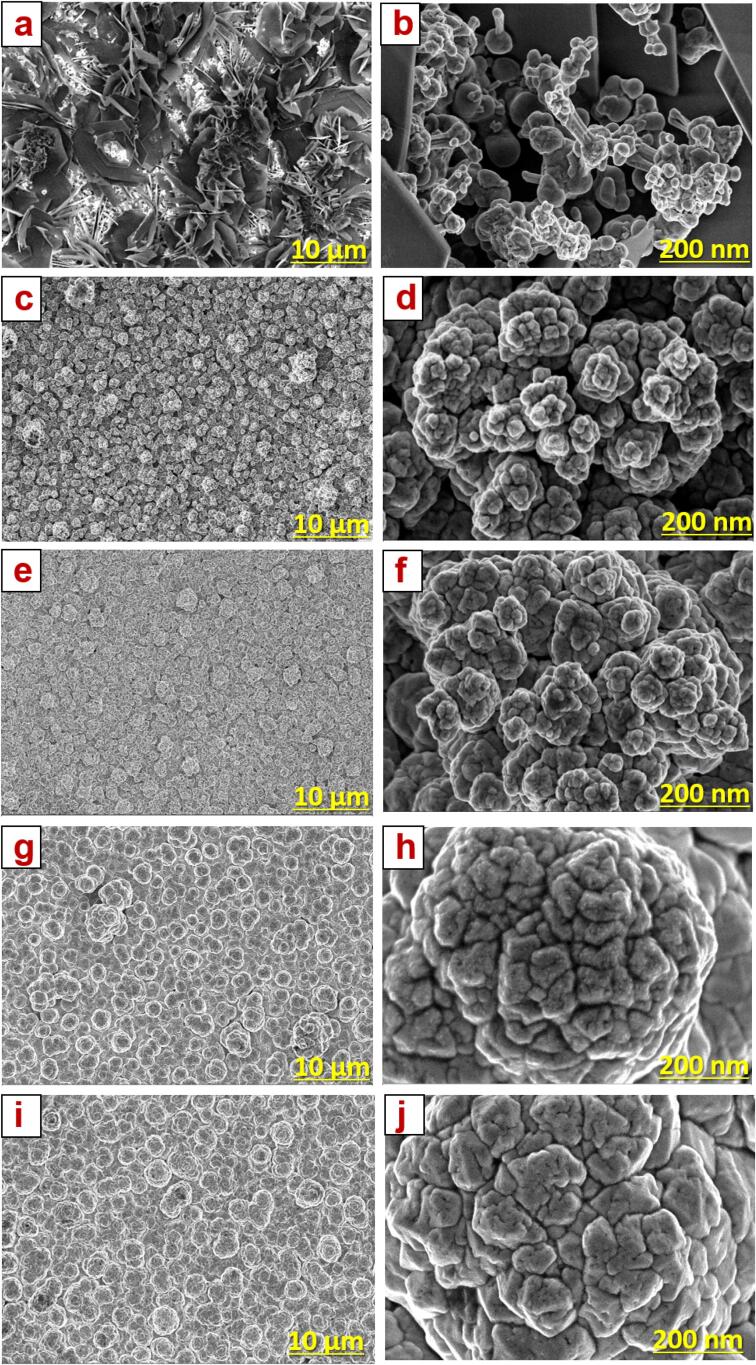
Surface morphology of Zn-Co films fabricated by (a, b) conventional method, (c, d) SC-CO_2_ @ 1500 psi, (e, f) SC-CO_2_ @ 2000 psi, (g, h) US-SC-CO_2_ @ 1500 psi, (i, j) US-SC-CO_2_ @ 2000 psi.

The Zn-Co film prepared by the conventional method was uneven with a very rough surface, which could be observed by bare eyes. In contrast, films prepared by SC-CO_2_ and US-SC-CO_2_ methods were exhibited very bright with a smoother surface. To validate the surface morphology and structure of the deposited metal ions, FE-SEM was explored. The observed images of all samples are presented in Fig. 2(a–j) with 10 µm and 200 nm magnifications. In the conventional method (Fig. 2a, b), the Zn-Co film was formed as rose petals like structure with the non-uniform distribution. It was reasonable because no more additives and levelers were used in this study, which results in uneven grain formation due to the H_2_ adsorption with metal ions reduction. The film prepared by the SC-CO_2_ method appeared as a cluster like a spherical structure with smaller grain size. It is due to the rapid formation of active metal sites at the electrode/electrolyte interface from the micelles generated by emulsified SC-CO_2_. Further, the elimination of H_2_ adsorption from the cathode surface was significantly enhanced by the non-polar CO_2_ at a supercritical state. Further, the increased pressure in the supercritical state from 1500 to 2000 psi, microbubbles explosion upstretched, reduces the grain size, and produces a compressed film as shown in Fig. 2(e, f). The observed results are revealed that the impact of pressure affected the nucleation growth and crystal size, which results in morphological transformation. Interestingly, ultrasonic agitation and supercritical state caused a much more compact and smoother film surface than SC-CO_2_ and conventional methods. It may be due to the fact of a prolonged supply of ultrasonic agitation generates a microjet that can helps to remove poorly adhered metal ions and H_2_ adsorption on the cathode [23], [33]. Moreover, this phenomenon intensely reduces the grain size, improves the adhesion property. From the effect of ultrasonic agitation, the reduced grains are combined and formed as a spherical nodule like structure (Fig. 2g–j).

**Fig. 3:**
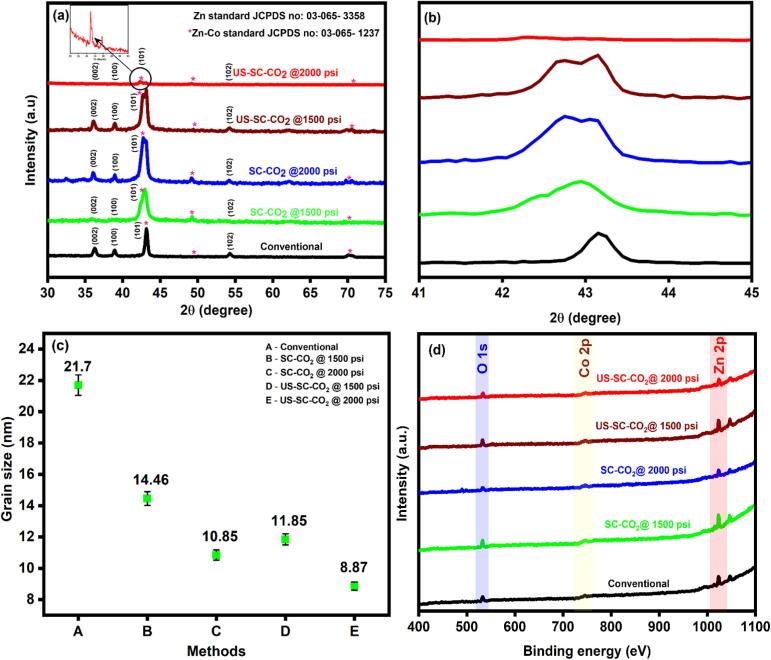
(a) XRD profile quantitative analysis spectrum, (b) magnified portion of main diffracted peak, (c) calculated grain size, (d) XPS survey spectrum fabricated films.

The cross-sectional images of the Zn-Co films were presented in Fig. S1(a–e). All the prepared films were distributed as a mixed metal/metal-hydroxide layer. It is noticed that the agglomerated particles present in the films indicate uneven crystallization, which is consistent with the poor diffraction peak of the equivalent XRD profile (Fig. 3a). In a conventional method, the layers were found to be two portions, such as (i) porous outer layer and (ii) inner barrier layer, which are caused for the increase in film thickness. Here the inner barrier layer only can contribute to the corrosion resistance. Because the outer layer suffers from the pores and lacks corrosion resistance (Fig. S1a). In contrast, stacked layers with refined particles were deposited in the SC-CO_2_ condition (Fig. S1b, c). Besides, an evenly deposited fine layer has resulted in the US-SC-CO_2_ condition (Fig. S1d, e). The use of ultrasonic waves eliminates the formation of stacked layers, forms the thin layer alone, and acts as an efficient corrosion inhibitor. Therefore, the application of electrodeposited films using US-SC-CO_2_ condition has a better surface morphology than any other method.

### 3.3 XRD and XPS analysis

Using XRD analysis, crystal structure and grain size were probed. The observed diffracted peaks for Zn-Co alloy films are shown in Fig. 3a. The peaks located at a diffracted angle 36.2°, 38.9°, 43.2°, and 54.3° were correspondingly indexed to the preferred orientation of (0 0 2), (1 0 0), (1 0 1), and (1 0 2) planes indicates the formation of hexagonal shaped metallic Zn (JCPDS no: 03-065-3358) [32], [38]. Compared to the conventional method, the peak shift was observed in SC-CO_2_ and US-SC-CO_2_ methods (Fig. 3b). The magnified portion of the main diffracted peak shows that the main peak shifted towards the left side from 43.2° to 42.9° and the peak gets broadening into a double-headed peak. It indicates that the zinc metal matrix was distorted with significant integration of cobalt [32]. Moreover, the peak broadening can be attributed to the grain refinement due to the emulsified CO_2_ well dispersed into the electrolyte, accelerating the metal ions through microbubble explosion, and significantly reducing the diffusion layer, and increases Co content during the deposition. In the US-SC-CO_2_ method, the main diffracted peak shifted towards 42.5° and relatively having lower intensity than other peaks. This further peak shift arises due to the increased amount of Co content in the film surface. Besides, the ultrasonic effect with SC-CO_2_ prominently enhances the cavitation behavior, increasing the cobalt content through the leveling effect; favors high energy and small atomic plane packing of deposited Zn. Furthermore, the ultrasonic agitation coarse the nucleation process and reduced the microstructure in crystal lattice causes grain refinement, resulting in peak intensity decrease and peak broadening. The average grain size of the Zn-Co films was calculated from the intense central peak using the Scherer equation, and they are illustrated in Fig. 3c. The US-SC-CO_2_ promotes high energy burst, which ultimately increases the Zn matrix’s cobalt content with reduced grain size.

To gain additional insight into the oxidation state of zinc and cobalt, XPS analysis was performed on the prepared films. The observed XPS survey of the fabricated films was presented in Fig. 3d. The survey spectrum shown in Fig. 3d specifies the presence of Zn, Co, and O from the as-prepared films and the absence of the additional impurities. The Co atom's small shoulder peak in the survey spectrums indicates that only a small proportion of Co has deposited in each film. However, compared to the conventional method, US-SC-CO_2_ assisted deposition method yielded more deposition of Co. As illustrated in Fig. S2(a, c, e, g, and i), the two major peaks at 1021.5 and 1046.1 eV can be attributed to the Zn 2p_3/2_ and Zn 2p_1/2_ of Zn(II). Further, the deconvoluted spectrum of Zn 2p_3/2_ gives a sharp peak (green color) at 1023 eV and a broad peak centered at 1021 eV (red color), indicates the presence of zinc in mixed oxidation states of Zn (II) and Zn (0), respectively. Additionally, the O 1s show the main peak at 532 eV, which is related to the metal hydroxyl group (M-OH) Fig. S2(b, d, f, h, and j).

### 3.4 Composition percentage in Zn-Co film

**Fig. 4:**
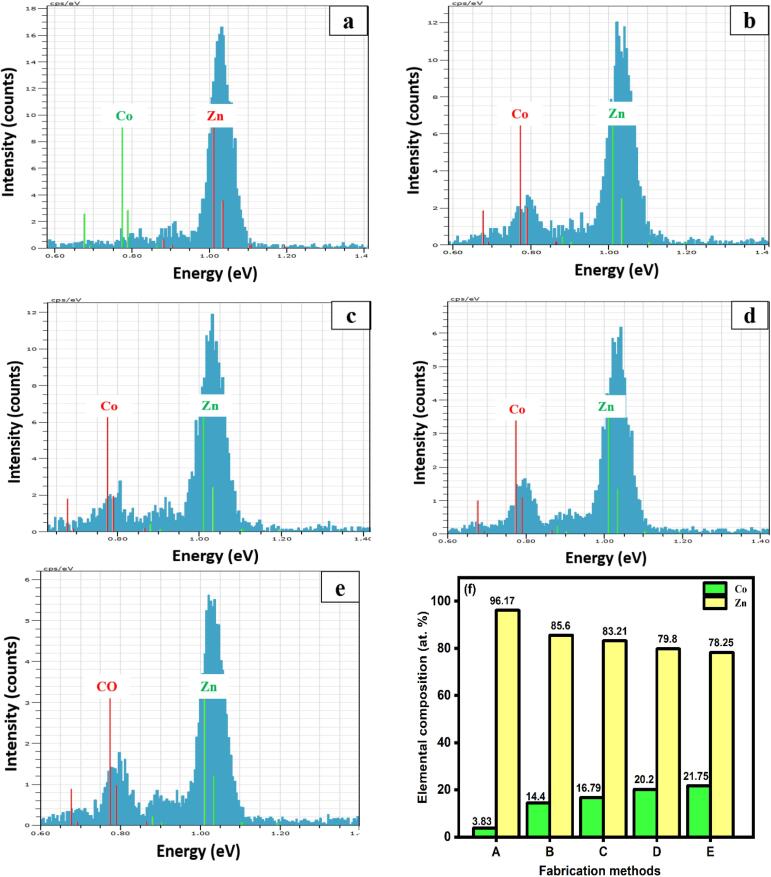
EDX profile quantitative analysis spectrum of prepared Zn-Co film by a) Conventional method, b) SC-CO_2_ 1500 psi, c) SC-CO_2_ 2000 psi, d) US-SC-CO_2_ 1500 psi, e) US-SC-CO_2_ 2000 psi, and f) average elemental percentage composition of fabricated films.

In order to verify the elemental percentage composition of the Zn-Co alloy ratio, EDX analysis was conducted for the fabricated films. The observed EDX profiles are shown in Fig. 4. The peaks appeared at 0.77, and 1.1 eV corresponds to Co and Zn elements, respectively. In Fig. 4a, the peak intensity of the Co element has weakly appeared, indicating a Co ratio is extremely low in the film prepared from the conventional method. During the conventional method reaction, the surface diffusion layer rapidly forms the thick Zn(OH)_2_ layer than the Co(OH)_2_ layer. Thus, the deposition magnitude of Zn ions was more than Co ions. But the trend changes in both typical SC-CO_2_ and US-SC-CO_2_ methods. In an SC-CO_2_ condition (Fig. 4(b, c)), the peak intensity of Co has been increased linearly over the conventional method (Fig. 4a), which means the pressurized-CO_2_ environment (1500 psi) produced microbubbles that accelerates the metal ions as well as decreases the thickness of Zn(OH)_2_ diffusion layer and relatively increased Co percentage of deposition. Compared to the 1500 psi, a further increase in pressure to 2000 psi leads to more Co ion deposition (Fig. 4c). On the other hand, with the introduction of US in SC-CO_2_ condition, the electrodeposition of Co content further gradually increases along with the similar trend in different pressures (as discussed in SC-CO_2_ condition) of 1500 psi (Fig. 4d) and 2000 psi (Fig. 4e). In contrast, upon increasing the Co content, the percentage of Zn content steadily decreases, and its results are displayed in (Fig. 4f). Furthermore, the observed results are in good agreement with XRD results. Thus, the results revealed that the US-SC-CO_2_-assisted electrodeposition could be an effective method than SC-CO_2_ and conventional methods for the Zn-Co thin film preparation.

### 3.5 Microhardness test

**Fig. 5:**
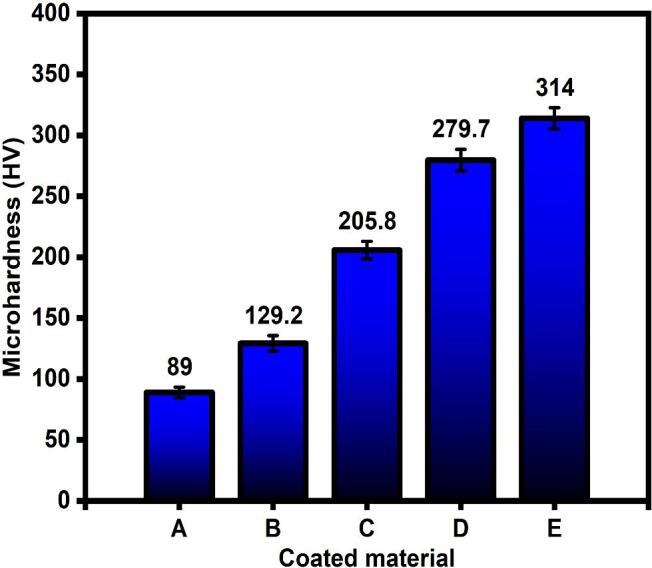
Microhardness of Zn-Co film prepared by A) Conventional method, B) SC-CO_2_ 1500 psi, C) SC-CO_2_ 2000 psi, D) US-SC-CO_2_ 1500 psi, E) US-SC-CO_2_ 2000 psi.

To investigate the mechanical property, Vicker’s hardness test was used to calculate the micro-hardness of the fabricated films. The recorded values are illustrated in Fig. 5. From this graph, all the films produced by SC-CO_2_ and US-SC-CO_2_ exhibits higher microhardness values. The film produced by the conventional method shows a lower microhardness value (~89 HV) due to the inhomogeneity of ions arrangements. Moreover, the cobalt ratio is relatively small in the conventional method. Whereas in SC-CO_2_ @ 1500 psi, the microhardness value increased to ~129.2 HV, and a further increase in pressure up to 2000 psi resulting in a higher microhardness value of ~205.8 HV. As discussed earlier, increasing the pressure decreases the Zn(OH)_2_ diffusion layer thickness and allows more Co ions to deposit onto the Zn Matrix. The increased proposition of Co ions enhanced hardness behavior. In US-SC-CO_2_, the well-known cavitation phenomenon is enhanced with increased pressure, which results in grain size refinement with compressed film and enhances the microhardness [23]. As a result, the SC-CO_2_ and inclusion of the ultrasonic agitation to the SC-CO_2_ process effectively progressed, yields refined crystallites with a homogenous surface. The adsorbed Co ions hinder the matrix grain boundary sliding resulting in enhanced mechanical strength with a higher microhardness value [7].

### 3.6 Corrosion studies

**Fig. 6:**
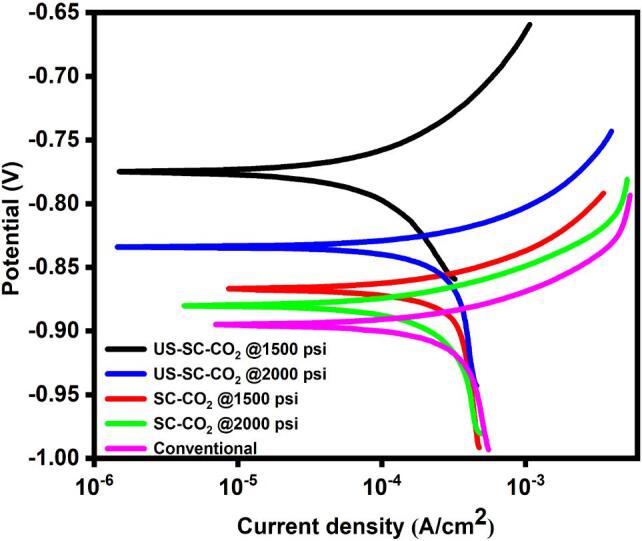
Tafel graph of the Zn-Co films.

The corrosion resistance of all the prepared Zn-Co films was evaluated through potentiodynamic polarization scanning (PPS) and electrochemical impedance spectroscopy (EIS) techniques in the presence of 3.5 wt.% NaCl solution as a corrosion solution. Fig. 6 shows the Tafel graph of the fabricated films. The estimated corrosion potentials (E_corr_) and the corrosion current (I_corr_) of the prepared films were calculated by the Tafel extrapolation method with the help of Nova software (version 2.1.4). The protective efficiency (8) was calculated from the corrosion current, and the results are presented in Table 1.

(8) $$Protectiveefficiency\left(PE\right)=\frac{I^{0}corr-I^{n}corr}{I^{0}corr}\times 100$$

where *I*^0^corr is conventional methods, and *I*^n^ corr is SC-CO_2_ and US-SC-CO_2_ methods. Based on polarization results, the best protective efficiency was observed in the order of US-SC-CO_2_ > SC-CO_2_ > conventional method. The addition of ultrasonic irradiation to the SC-CO_2_ electrodeposition approach resulted in reduced grain size of coatings and improved surface characteristics. Therefore, the US-SC-CO_2_ electrodeposition process’s application significantly enhanced the corrosion resistance of prepared films [34]. Further, the influence of supercritical pressure on corrosion resistance of the prepared films was also performed. Compared to the 1500 psi pressure, the films prepared in 2000 psi pressure expose lower corrosion resistance. This contrary effect may affirm from the increased distribution of grain boundaries with reduced grain size. Thus, the grain boundaries are mightily reactive than the grain matrix, which leads to intergranular corrosion [23]. Therefore, the US-SC-CO_2_ @ 1500 psi process achieved the highest protective efficiency of 73.7 5%.

**Table 1:** polarization results of prepared films.

Methods	E_corr_ (v)	I_corr_ (μA)	Protective efficiency (%)
Conventional	−0.9	48.22	–
SC-CO_2_ @1500 psi	−0.86	27.29	43.41
SC-CO_2_ @2000 psi	−0.88	29.43	38.97
US-SC-CO_2_ @1500 psi	−0.77	12.66	73.75
US-SC-CO_2_ @2000 psi	−0.83	24.96	48.24

**Fig. 7:**
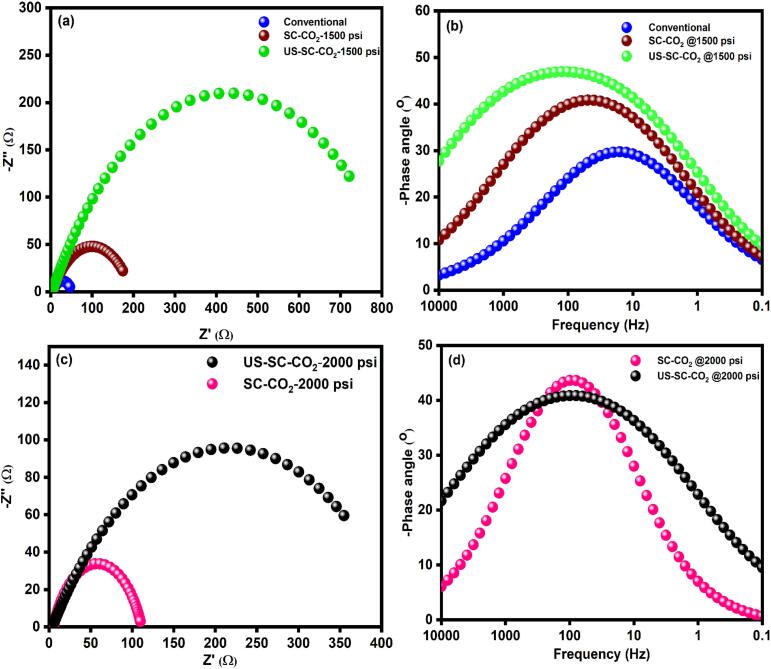
Impedance results of the Zn-Co films at 0 h. (a, c) Nyquist plot (b, d) bode plot.

**Fig. 8:**
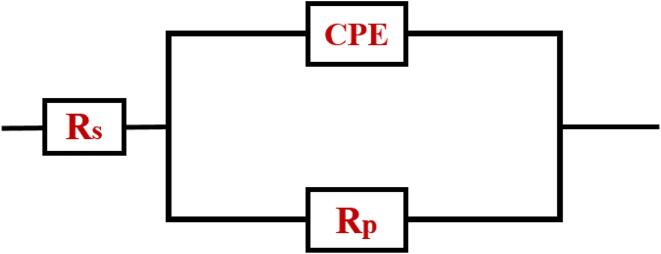
Electrical equivalent circuit model.

**Fig. 9:**
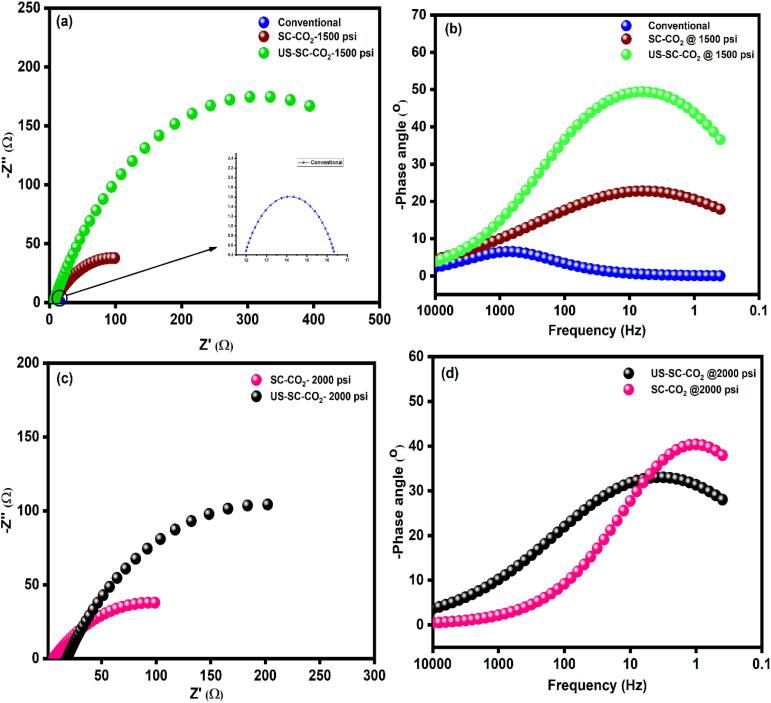
Impedance results of the Zn-Co films at 24 h. (a, c) Nyquist plot (b, d) bode plot.

Further to validate the Tafel graph, EIS was performed for all the prepared films. Fig. 7(a–d) presents the Nyquist and Bode plot of the prepared Zn-Co films. The obtained EIS data were well fitted one time constant with the equivalent electrical circuit (EEC) model illustrated in Fig. 8. Where R_s_ denotes the solution’s resistance, R_p_ indicates polarization resistance, and CPE is denoted as a constant phase element, respectively. The double-layer capacitance (C_dl_) depends upon the frequency module. In corrosion studies, C_dl_ cannot be denoted with a pure capacitor due to the frequency dispersion effect. Thus, it can be denoted as a constant phase element (CPE). As displayed in Fig. 7, all the fabricated Zn-Co films are exhibiting a depressed semicircle loop with a different radius in the Nyquist plot, and a hump-like shape appeared in the bode plot. From the semicircle loop, the polarization resistance (R_p_) was calculated. The higher R_p_ value denotes high corrosion resistance. The shapes of the respective Nyquist plot shows some differences for 0 and 24 h of submerging time in corrosion solution. Fig. 7(a, c) shows a well-defined semicircle loop in the Nyquist plot, and Fig. 7(b, d) shows a well-defined hump in the Bode plot at 0 hr immersion. The 24 h immersed films depicted a distorted semicircle loop in Fig. 9(a, b), which attributes the film dissolution, poor surface stability, and micro-porous nature. The fitted impedance results were presented in Tables 2 and 3.

**Table 2:** Impedance analysis data of 0 h immersion.

Methods	R_s_ (Ω)	R_p_ (Ω)	CPE (Ω)
Conventional	4.463	47.325	4.99 × 10^−2^
SC-CO_2_ @1500 psi	4.205	191.89	1.24 × 10^−2^
SC-CO_2_ @2000 psi	5.301	134.03	4.35 × 10^−3^
US-SC-CO_2_ @1500 psi	3.640	848.14	3.8 × 10^−3^
US-SC-CO_2_ @2000 psi	3.852	424.82	8.36 × 10^−3^

**Table 3:** Impedance analysis data after 24 h immersion.

Methods	R_s_ (Ω)	R_p_ (Ω)	CPE (Ω)
Conventional	11.712	4.88	4.46 × 10^−2^
SC-CO_2_ @1500 psi	5.689	178.94	6.6 × 10^−2^
SC-CO_2_ @2000 psi	10.502	156.06	5.8 × 10^−2^
US-SC-CO_2_ @1500 psi	6.093	624.05	1.48 × 10^−2^
US-SC-CO_2_ @2000 psi	18.099	364.16	3.6 × 10^−2^

From the EIS results, it was evident that the film prepared in

US-SC-CO_2_ electrodeposition method succeeds in higher corrosion resistance than other methods. In detail, the films prepared by SC-CO_2_ have a relatively higher cobalt ratio than the conventional method. It is expected that the increase in Co content in Zn matrix can possibly provide a surface protection to sacrificial film (Zn) though a shielding effect. Thus, the corrosion on the film’s surface prepared through SC-CO_2_ occurs four times slower than conventional. In US-SC-CO_2_, the increased proportion of cobalt ions in the zinc matrix enhances the films’ corrosion resistance behavior over the Cl^−^ ions. Thus the US-SC-CO_2_ shows higher corrosion resistance of 18 times greater than conventional and four times higher than usual SC-CO_2_ methods.

Corrosion protection is mainly developed by inhibitor adsorption in either physisorption or metal surface chemisorption, which results in the formation of protective layers. Zn corrosion can be defined as occurring with the following two consecutive one-electron transfer reactions mechanism [39].

(9) Zn (s) → Zn^+^ (ads) + e^–^

(10) Zn^+^ (ads) → Zn^2+^ (ads) + e^–^

(11) Zn^2+^ (ads) → Zn^2+^ (aq)

During the initial phases of corrosion, zinc preferably dissolves rapidly, whereas cobalt rather slowly dissolves. The rapid dissolution of zinc in corrosion solution related to the coverage relaxation of adsorbed ions resulting in the formation of different corrosion products, such as zinc hydroxide (Zn(OH)_2_), zinc oxide (ZnO), and zinc hydroxyl chloride (Zn_5_(OH)_8_Cl_2_·H_2_O) [39]. The Zn–Co coating's dezincification leads to an improved barrier layer by the incorporated Co ions into the Zn matrix act as a passive layer that slow down the corrosion, protect the films’ surface, and reduces the corrosion rate. Thus, the complex composition of the protective layer covers the surface, and as a result, electrochemical corrosion is retarded. The formation of the shielding layer on the films’ surface did not show evidence of passivation. This passivation behavior is in accordance with the literature [14], [32], [33], [39], [40], [41], [42], [43]. The possible reasons to validate this corrosion phenomenon are compact smoother surface, refined grain size, an increased proportion of cobalt ions on the films’ surface. However, the observation was extended with SC-CO_2_ and US-SC-CO_2_ at an increased pressure of 2000 psi. However, as mentioned earlier, the increased pressure significantly enhanced the cavitation behavior resulting in further grain refinement with more grain nodules. The formed grain nodules with further refined grains lead to intergranular corrosion. These EIS results resemble Tafel observations.

### 3.7 Morphology analysis after corrosion

**Fig. 10:**
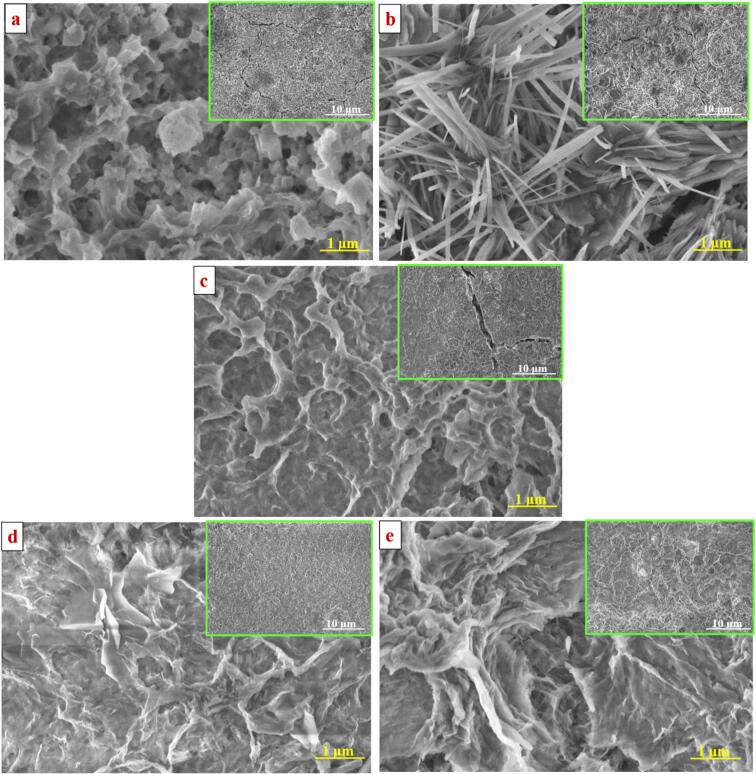
Surface morphology of Zn-Co films after corrosion a) Conventional method, b) SC-CO_2_ @ 1500 psi, c) SC-CO_2_ @ 2000 psi, d) US-SC-CO_2_ @ 1500 psi, e) US-SC-CO_2_ @ 2000 psi.

After the corrosion reaction, the samples were probed with morphological studies through FE-SEM to validate the above polarization resistance discussion. Fig. 10 presents the surface morphology of all the prepared samples after corrosion analysis. As seen in Fig. 10, it can be seen that the entire morphology of the prepared films was lost its original structure after corrosion. From the results, the conventional method shows an inferior surface after corrosion. The presence of rose petal-like structures is vulnerably encountered by Cl^−^ ions (Fig. 10a). Moreover, the micro-cracks and pores were more abundant on the film surface due to ions’ lower hardness and arrangement. In the SC-CO_2_ method (Fig. 10b and c), the micro-cracks and pores are comparatively lesser than the conventional method. The provision of metal ions is denser with an increased proportion of cobalt content than the conventional method. On the other hand, the US-SC-CO_2_ electrodeposition application shows a smoother surface than conventional and SC-CO_2_ methods (Fig. 10d and e). There are no evident micro cracks observed. The US-SC-CO_2_ electrodeposited films have relatively increased cobalt content than other methods were actively protecting against the Cl^−^ ions. The pressure increased to 2000 psi in SC-CO_2_ and US-SC-CO_2,_ having a more tarnished surface than 1500 psi of SC-CO_2_ and US- SC-CO_2_. It is well known that the increased pressure simultaneously increases the microbubble explosion, which significantly reduced the grain size that is slightly reactive to the Cl^−^ ions [23]. The observed morphology analysis resembles with the electrochemical evaluations. Thus, the optimum pressure to fabricate the Zn-Co metal films has been suggested to 1500 psi.

## 4 Conclusions

The fabrication of Zn-Co film by novel US-SC-CO_2_ electrodeposition method was demonstrated in this work. The Zn-Co film prepared by the US-SC-CO_2_ electrodeposition method exhibits a smoother and brighter surface, smaller grain size, and more compact microstructure than the films prepared by conventional and SC-CO_2_ methods. Based on the FE-SEM results, the surface morphology is examined and shown that as pressure increases, the surface homogeneity also increases with reduced grain size. Moreover, EDX confirms that the Co content in the films is increased with increased pressure, and thus, increased microhardness. The influence of ultrasound with the SC-CO_2_ electrodeposition method had reduced the intensity of peak with a broad spectrum, which means the deposited crystal size shrinks due to the strength of applied ultrasonic irradiation. From the grain size calculation, it was apparent to know that the films prepared by US-SC-CO_2_ achieve a smaller grain size (i.e., US-SC-CO_2_ @ 2000 psi = 8.87 nm; US-SC-CO_2_ @ 1500 psi = 11.85 nm) than that of SC-CO_2_ and conventional methods. Based on the corrosion evaluation, the US-SC-CO_2_ approach is found to be highly efficient than that of other methods by 73.75%. However, the increased pressure (2000 psi) refines the grain size, and it slightly decreases the polarization resistance than 1500 psi pressure. The results conclude that increased pressure helps to enhance the mechanical property but suffers in polarization resistance. Therefore, the application of the US-SC-CO_2_ electrodeposition approach suggests that the optimum parameter is 1500 psi @ 42 kHz/ 20 W cm^−2^, which is favorable to fabricate Zn-Co composite film with enhanced properties.

## CRediT authorship contribution statement

**Pandiyarajan Sabarison:** Conceptualization, Methodology, Software, Investigation, Writing - original draft. **Ganesan Muthusankar:** Data curation, Formal analysis, Visualization, Writing - review & editing. **Ai-Ho Liao:** Writing - review & editing. **Manickaraj Shobana Sebastin Mary:** Data curation, Formal analysis, Visualization, Writing - review & editing. **Sheng-Tung Huang:** Resources, Supervision. **Ho-Chiao Chuang:** Resources, Supervision, Writing - review & editing.

## Declaration of Competing Interest

The authors declare that they have no known competing financial interests or personal relationships that could have appeared to influence the work reported in this paper.

## References

[1] Dwivedi D., Lepková K., Becker T. (2017). Carbon steel corrosion: a review of key surface properties and characterization methods. RSC Adv..

[2] Youssef K.M.S., Koch C.C., Fedkiw P.S. (2004). Improved corrosion behavior of nanocrystalline zinc produced by pulse-current electrodeposition. Corros. Sci..

[3] Li B., Zhang W. (2020). Facile synthesis and electrochemical properties of a novel Ni-B/TiC composite coating via ultrasonic-assisted electrodeposition. Ultrason. Sonochem..

[4] Tiétcha G.F., Mears L.L.E., Dworschak D., Roth M., Klüppel I., Valtiner M. (2020). Adsorption and diffusion moderated by polycationic polymers during electrodeposition of zinc. ACS Appl. Mater. Interfaces.

[5] Hu C., Xie X., Zheng H., Qing Y., Ren K. (2020). Facile fabrication of superhydrophobic zinc coatings with corrosion resistance via an electrodeposition process. New J. Chem..

[6] Hasanpour P., Salehikahrizsangi P., Raeissi K., Santamaria M., Calabrese L., Proverbio E. (2019). Dual Ni/Ni-Co electrodeposited coatings for improved erosion-corrosion behaviour. Surf. Coat. Technol..

[7] He T., He Y.i., Li H., Su Z., Fan Y.i., He Z.e. (2018). Fabrication of Ni-W-B_4_C composite coatings and evaluation of its micro-hardness and corrosion resistance properties. Ceram. Int..

[8] Et Taouil A., Mahmoud M.M., Lallemand F., Lallemand S., Gigandet M.-P., Hihn J.-Y. (2012). Corrosion protection by sonoelectrodeposited organic films on zinc coated steel. Ultrason. Sonochem..

[9] Abioye O.P., Musa A.J., Loto C.A., Fayomi O.S.I., Gaiya G.P. (2019). Evaluation of corrosive behavior of zinc composite coating on mild steel for marine applications. J. Phys. Conf. Ser..

[10] Almeida M., Rovere C., Lima L., Ribeiro D., Souza C. (2019). Glycerol effect on the corrosion resistance and electrodeposition conditions in a zinc electroplating process. Mater. Res..

[11] Bahrololoom M.E., Gabe D.R., Wilcox G.D. (2003). Development of a bath for electrodeposition of zinc-cobalt compositionally modulated alloy multilayered coatings. J. Electrochem. Soc..

[12] Bajat J.B., Miskovic-Stankovic V.B., Maksimović M.D., Dražić D.M., Zec S. (2002). Electrochemical deposition and characterization of Zn-Co alloys and corrosion protection by electrodeposited epoxy coating on Zn-Co alloy. Electrochim. Acta.

[13] Mouanga M., Ricq L., Bercot P. (2008). Electrodeposition and characterization of zinc–cobalt alloy from chloride bath; influence of coumarin as additive. Surf. Coat. Technol..

[14] Kahoul A., Azizi F., Bouaoud M. (2017). Effect of citrate additive on the electrodeposition and corrosion behaviour of Zn–Co alloy. Trans. IMF.

[15] Zhang X., Heinonen S., Levänen E. (2014). Applications of supercritical carbon dioxide in materials processing and synthesis. RSC Adv..

[16] Bartlett P.N., Cook D.A., George M.W., Hector A.L., Ke J., Levason W., Reid G., Smith D.C., Zhang W. (2014). Electrodeposition from supercritical fluids. Phys. Chem. Chem. Phys..

[17] Luo X., Chen C.-Y., Chang T.-F.-M., Hosoda H., Sone M. (2015). Crystal growth of cobalt film fabricated by electrodeposition with dense carbon dioxide. J. Electrochem. Soc..

[18] Shinoda N., Shimizu T., Chang T., Shibata A., Sone M. (2013). Cu electroplating using suspension of supercritical carbon dioxide in copper-sulfate-based electrolyte with Cu particles. Thin Solid Films.

[19] Chang T., Shimizu T., Ishiyama C., Sone M. (2013). Effects of pressure on electroplating of copper using supercritical carbon dioxide emulsified electrolyte. Thin Solid Films.

[20] Chuang H.C., Lai W.H., Sanchez J. (2015). An investigation of supercritical-CO_2_ copper electroplating parameters for application in TSV chips Related content. J. Micromech. Microeng..

[21] Gao H., Zhu K., Hu G., Xue C. (2017). Large-scale graphene production by ultrasound-assisted exfoliation of natural graphite in supercritical CO_2_/H_2_O medium. Chem. Eng. J..

[22] Gao H., Xue C., Hu G., Zhu K. (2017). Production of graphene quantum dots by ultrasound-assisted exfoliation in supercritical CO_2_/H_2_O medium. Ultrason. Sonochem..

[23] Chuang H., Yang H., Wu G., Sanchez J., Shyu J. (2018). The effects of ultrasonic agitation on supercritical CO_2_ copper electroplating. Ultrason. Sonochem..

[24] Lee D., Gan Y., Chen X., Kysar J. (2007). Influence of ultrasonic irradiation on the microstructure of Cu/Al_2_O_3_, CeO_2_ nanocomposite thin films during electrocodeposition. Mater. Sci. Eng. A.

[25] Theerthagiri J., Madhavan J., Lee S.J., Choi M.Y., Ashokkumar M., Pollet B.G. (2020). Sonoelectrochemistry for energy and environmental applications. Ultrason. Sonochem..

[26] Pollet B.G., Hihn J.-Y., Doche M.-L., Lorimer J.P., Mandroyan A., Mason T.J. (2007). Transport limited currents close to an ultrasonic horn: equivalent flow velocity determination. J. Electrochem. Soc..

[27] Pollet B.G. (2012). Power Ultrasound in Electrochemistry.

[28] Hihn J.-Y., Doche M.-L., Mandroyan A., Hallez L., Pollet B.G. (2011). Respective contribution of cavitation and convective flow to local stirring in sonoreactors. Ultrason. Sonochem..

[29] Pollet B., Ashokkumar M. (2019). Introduction to Ultrasound, Sonochemistry and Sonoelectrochemistry.

[30] Hihn J.-Y., Doche M.-L., Hallez L., Et Taouil A., Pollet B.G., Sonoelectrochemistry (2018). Both a tool for investigating mechanisms and for accelerating processes. Electrochem. Soc. Interface.

[31] Kafashan H., Azizieh M., Vatan H. (2016). Ultrasound-assisted electrodeposition of SnS: Effect of ultrasound waves on the physical properties of nanostructured SnS thin films. J. Alloys Compd..

[32] Riđošić M., García-Lecina E., Salicio-Paz A., Bajat J. (2020). The advantage of ultrasound during electrodeposition on morphology and corrosion stability of Zn-Co alloy coatings The advantage of ultrasound during electrodeposition on morphology and corrosion stability of Zn-Co alloy coatings. Trans. IMF..

[33] Kobayashi K., Chiba A., Minami N. (2000). Effects of ultrasound on both electrolytic and electroless nickel depositions. Ultrasonics.

[34] Chuang H.C., Jiang G.W., Sanchez J. (2020). Study on the changes of ultrasonic parameters over supercritical Ni-Co electroplating process. Ultrason. Sonochem..

[35] Gao H., Hu G., Liu K., Wu L. (2017). Preparation of waterborne dispersions of epoxy resin by ultrasonic-assisted supercritical CO_2_ nanoemulsification technique. Ultrason. Sonochem..

[36] Luo D., Qiu T., Lu Q. (2007). Ultrasound-assisted extraction of ginsenosides in supercritical CO_2_ reverse microemulsions. J. Sci. Food Agric..

[37] Safavi M.S., Tanhaei M., Ahmadipour M.F., Adli R.G., Mahdavi S., Walsh F.C. (2020). Electrodeposited Ni-Co alloy-particle composite coatings: a comprehensive review. Surf. Coat. Technol..

[38] Chen Y., Schneider P., Liu B.J., Borodin S., Ren B., Erbe A. (2013). Electronic structure and morphology of dark oxides on zinc generated by electrochemical treatment. Phys. Chem. Chem. Phys..

[39] Lima-Neto P.D., Correia A.N., Colares R.P., Araujo W.S. (2007). Corrosion study of electrodeposited Zn and Zn-Co coatings in chloride medium. J. Braz. Chem. Soc..

[40] Sriraman K.R., Brahimi S., Szpunar J.A., Osborne J.H., Yue S. (2013). Characterization of corrosion resistance of electrodeposited Zn–Ni Zn and Cd coatings. Electrochim. Acta.

[41] Tafreshi M., Allahkaram S.R., Farhangi H. (2016). Comparative study on structure, corrosion properties and tribological behavior of pure Zn and different Zn-Ni alloy coatings. Mater. Chem. Phys..

[42] Rashmi S., Elias L., Hegde A.C. (2017). Multilayered Zn-Ni alloy coatings for better corrosion protection of mild steel. Eng. Sci. Technol. an Int. J..

[43] Leidheiser H., Suzuki I. (1981). Cobalt and nickel cations as corrosion inhibitors for galvanized steel. J. Electrochem. Soc..

